# The impact of the opioid crisis on U.S. state prison systems

**DOI:** 10.1186/s40352-021-00143-9

**Published:** 2021-07-24

**Authors:** Christy K. Scott, Michael L. Dennis, Christine E. Grella, Allison F. Mischel, John Carnevale

**Affiliations:** 1 Chestnut Health Systems, 221 W. Walton St, Chicago, IL 60610 USA; 2 Chestnut Health Systems, 448 Wylie Dr, Normal, IL 61761 USA; 3 Carnevale Associates LLC, 4 Belinder Rd, Gaithersburg, MD 20878 USA

**Keywords:** Opioid use disorder (OUD), Medications for opioid use disorder (MOUD), State prison systems, OUD service cascade

## Abstract

**Background:**

Prior studies have documented limited use of medications to treat opioid use disorders (OUD) for people incarcerated within state prisons in the United States. Using the framework of the criminal justice OUD service cascade, this study interviewed representatives of prison systems in states most heavily impacted by opioid overdose regarding the provision of medications for OUD (MOUD).

**Methods:**

A stratified sampling strategy included states with high indicators of opioid-overdose deaths. Two sampling strata targeted states with: 1) OUD overdose rates significantly higher than the per capita national average; or 2) high absolute number of OUD overdose fatalities. Interviews were completed with representatives from 21 of the 23 (91%) targeted states in 2019, representing 583 prisons across these states. Interviews assessed service provision across the criminal justice OUD service cascade, including OUD screening, withdrawal management, MOUD availability and provision, overdose prevention, re-entry services, barriers, and needs for training and technical assistance.

**Results:**

MOUD (buprenorphine, methadone, or naltrexone) was available in at least one prison in approximately 90% of the state prison systems and all three medications were available in at least one prison in 62% of systems. However, MOUD provision was limited to subsets of prisons within these systems: 15% provided buprenorphine, 9% provided methadone, 36% provided naltrexone, and only 7% provided all three. Buprenorphine and methadone were most frequently provided to pregnant women or individuals already receiving these at admission, whereas naltrexone was primarily used at release. Funding was the most frequently cited barrier for all medications.

**Conclusion:**

Study findings yield a complex picture of how, when, and to whom MOUD is provided across prisons within prison systems in states most heavily impacted by opioid overdose in the United States and have implications for expanding availability.

**Supplementary Information:**

The online version contains supplementary material available at 10.1186/s40352-021-00143-9.

## Introduction

Based on the 2016 National Survey on Drug Use and Health (NSDUH), 1 in 4 people with an opioid use disorder (OUD) had contact with some part of the criminal justice system in the preceding year. Furthermore, these individuals have significantly higher rates of OUD than those without criminal justice involvement (8.5% vs. 0.8%; Substance Abuse and Mental Health Services Administration [SAMHSA], 2017). Moreover, the rates of lifetime OUD increase as people penetrate further into the justice system (e.g., arrest, community supervision, county jails, and state prisons; SAMHSA, 2017). Similarly, another study using the NSDUH data from 2015 to 2016 found that the likelihood of criminal justice involvement increased with greater severity of opioid use (Winkelman, Chang, & Binswanger, 2018). Severity of use was defined as progressing from prescription opioid use/misuse/disorder to heroin use, based on epidemiological studies on patterns of use over time (Alpert, Powell, & Pacula, 2018; Cicero, Ellis, & Haney, 2017; Compton, Jones, & Baldwin, 2016). Opioid use severity was strongly associated with increases in criminal justice involvement, even after controlling for socio-demographics, mental and physical health problems, and other substance use (Winkelman et al., 2018). Combined, these studies clearly document the inter-relationships between justice involvement and opioid use.

In addition to this relationship, the high risk of overdose following incarceration demands attention. Even after years of incarceration, people with OUD are at high risk of opioid relapse and overdose, as well as recidivism upon community re-entry (Binswanger, Nguyen, Morenoff, Xu, & Harding, 2020; Bukten et al., 2017; Keen, Young, Borschmann, & Kinner, 2020; Winter et al., 2015). The risk of death from opioid overdose is particularly high in the immediate period following release from prison, with one study showing a 40-fold increase in risk of opioid overdose fatality among individuals in the two weeks following their release from prison, compared with the general population (Ranapurwala et al., 2018).

### Effects of MOUD treatment on post-release outcomes

Several studies have assessed the impact of medication for opioid use disorder (MOUD) treatment on post-release relapse, criminal re-offending, and re-incarceration. There is considerable evidence that treatment with opioid agonists for people while incarcerated or prior to their release has beneficial effects. A recent meta-analysis of 4 randomized controlled trials (RCTs) involving 807 incarcerated individuals examined outcomes of methadone treatment delivered in prison (Moore et al., 2019). The meta-analysis showed that methadone provided during incarceration increased community treatment engagement (*n* = three studies; odds ratio [OR] = 8.69), and reduced both illicit opioid use (*n* = four studies; OR = 0.22) and injection drug use (*n* = three studies), but did not significantly reduce recidivism. Another review of RCTs demonstrated that initiating opioid agonist treatment during incarceration versus after release was associated with higher rates of entry into community treatment and reduced heroin use (Sharma et al., 2016). In one study, individuals who continued treatment with methadone post-release had a 65% lower risk of returning to custody than a group that terminated treatment post-release and a group of non-methadone controls with OUD (Farrell-MacDonald, MacSwain, Cheverie, Tiesmaki, & Fischer, 2014). In another RCT, individuals who initiated methadone treatment and counseling pre-release were more likely to continue treatment post-release, and had lower rates of heroin/opioid use and re-offending over 6 months compared with individuals who received counseling only (Gordon, Kinlock, Schwartz, & O’Grady, 2008).

Similarly, individuals who initiated treatment with buprenorphine while incarcerated were more likely to enter community-based SUD treatment post-release (Gordon et al., 2014), and to receive more days of buprenorphine treatment in community treatment programs over the 12-months post-release, but it was not associated with better outcomes in terms of heroin and cocaine use and criminal behavior relative to participants who initiated buprenorphine treatment after release (Gordon et al., 2017).

Studies examining the effects of naltrexone (an opioid antagonist) on post-release outcomes are mixed. In a multi-site RCT of extended-release naltrexone injection versus treatment-as-usual (i.e., counseling and referral to community treatment at discharge), Lee et al. (2016) found that justice-involved individuals in the naltrexone group had lower rates of and time to relapse at 24 weeks than those in the control group; however, there were no differences in opioid-positive urine tests at approximately one year after the treatment phase (Lee et al., 2016). Additionally, in a pilot study conducted with incarcerated individuals prior to their release, those who initiated treatment with naltrexone and who received at least six injections were significantly less likely to have positive tests for opioids or to be rearrested compared to those who received less than six injections (Gordon et al., 2015).

Despite these studies demonstrating the generally beneficial effects of MOUD treatment on reducing relapse and recidivism among justice-involved individuals and strong recommendations for its use in criminal justice settings (Chandler, Fletcher, & Volkow, 2009), there is a paucity of information about what is available, accessible, and utilized throughout the criminal justice system. Reliable information is urgently needed regarding the current strategies for addressing OUD and risk of opioid overdose, the extent to which these strategies are implemented, and the steps necessary to mitigate the opioid crisis within the justice system. The current study builds upon prior surveys of the provision of MOUD within correctional systems, and extends this research by incorporating more in-depth questions regarding how medications are provided (e.g., timing, eligibility, duration). This study also addresses the barriers encountered to the provision of MOUD and needs for training and technical assistance in order to assess the current policies and practices within state prison systems on treatment for OUD.

### Prior studies on provision of MOUD in U.S. prisons

Several prior surveys have examined the provision of medications and other forms of treatment for OUD within prisons and other correctional settings. In a 2003 survey of U.S. state and federal prison medical directors, less than half (*n* = 19/40, 48%) of respondents reported their system provided methadone detoxification or maintenance services. In most cases (13/19, 68%) where methadone maintenance was provided, it was available only to opioid-dependent pregnant women (Rich et al., 2005). Respondents most often cited logistical obstacles and security concerns as barriers, as well as administrative opposition to the use of methadone stemming from a preference for abstinence-based treatment. Moreover, only three of 40 respondents (8%) reported that their system referred individuals to methadone treatment in the community upon release.

In 2008, a similar study surveyed prison medical directors in all 50 states, the District of Columbia, and the Federal Department of Corrections. Of the 40 respondents, 28 prison systems (55%) offered limited access to methadone and 7 systems (14%) offered limited access to buprenorphine (Nunn et al., 2009; Nunn, Zaller, Dickman, Nijhawan, & Rich, 2010). One-half of the systems provided methadone only to pregnant women or individuals with chronic pain. Referral to community-based methadone treatment upon release remained low (45%) but, unlike in prior studies, now also included referral to buprenorphine providers (29%). Respondents indicated the lack of MOUD implementation stemmed from the preference for “drug-free” detoxification and treatment services, including staff resistance to use of medications (Nunn et al., 2009; Nunn et al., 2010; Nunn & Zaller, 2009). Limited partnerships with community providers was also cited as a barrier to post-release referrals.

In a comprehensive survey of 50 correctional agencies in the U.S. (representing a cross-section of prisons, jails, drug court, probation and parole) conducted as part of the Criminal Justice Drug Abuse Treatment Services (CJ-DATS) cooperative agreement, treatment with MOUD was most often restricted to pregnant women, individuals in withdrawal, and HIV+ individuals (Friedmann et al., 2012). As in prior surveys, referral to community-based MOUD providers was low (8%) and the primary barriers to MOUD utilization stemmed from a preference for drug-free treatment, limited knowledge of the benefits of MOUD, security concerns, regulations prohibiting use of MOUD for certain agencies, and the lack of qualified medical staff (Friedmann et al., 2012).

Despite these pervasive barriers to use of MOUD, a majority of respondents in the CJ-DATS sample stated they were “open” to the possibility of expanding or introducing treatment with methadone, buprenorphine, and naltrexone in the future. Similarly, correctional agents who had observed positive effects of MOUD treatment upon individuals they supervised also expressed more favorable attitudes (Mitchell et al., 2016). Given the concentration of individuals with OUD in the criminal justice system, there is an opportunity to expand the range of interventions provided to address the high risk of opioid overdose and fatality among this population, including screening, treatment with MOUD, overdose prevention, re-entry services, and linkage to community-based providers (Brinkley-Rubinstein et al., 2018).

### Current study

The current study builds on this foundation of prior research on the extent to which MOUD is provided in United States prisons within the context of the current opioid epidemic. The interview questions were guided by the OUD service cascade, which is a framework for evaluating the gaps in service delivery across a continuum of services, from screening, referral to treatment, treatment engagement, and continuing care (Socías et al., 2018; Williams, Nunes, Bisaga, & Olfson, 2018; Williams, Nunes, & Olfson, 2017). Given the critical issue of transition and linkage to community-based treatment for OUD following prison discharge, as well as the extremely high risk of opioid overdose during this transition period, as noted previously, we modify the OUD cascade model to incorporate a focus on overdose prevention (Wakeman, McKenzie, Jeronimo, & Rich, 2009) and community re-entry services, consistent with other criminal justice-focused service cascade models (Belenko et al., 2017; National Governors Association & American Correctional Association, 2021). Moreover, to situate the findings within the broader context of the opioid epidemic, the sampling strategy selected states that have been most impacted by the opioid crisis on the basis of either a high population prevalence of opioid overdose deaths or a high number of opioid overdose fatalities.

## Methods

### Overview

The goal of this project was to interview prison system officials in states severely impacted by the opioid epidemic in the United States in order to understand how these systems have responded to the opioid crisis. These interviews asked about the availability and implementation of each part of the OUD service cascade including: 1) opioid withdrawal management; 2) screening and assessment to identify opioid use problems; 3) MOUD availability and provision, including barriers to implementation; 4) overdose prevention; and 5) re-entry planning. Lastly, participants were asked their opinions about how to better address the opioid crisis within state prisons, specifically regarding the need for training and resources.

In 2019, structured interviews were completed with representatives from 21 of 23 (91%) targeted prison systems representing states most impacted by the current opioid overdose crisis. (The state sampling design is detailed below.) Interviews were conducted with the state correctional commissioner and/or their designees; participation was voluntary and interviewees were not compensated. The study obtained a “Certificate of Confidentiality” from the National Institutes of Health and was conducted under the supervision of Chestnut Health Systems’ Institutional Review Board for the Protection of Human Subjects.

### State selection

As in other drug-related deaths, opioid-overdose deaths are not randomly distributed across the general population (Monnat, 2018). Epidemiological analyses of the geographic distribution of opioid-related deaths show distinctive patterns related to urbanicity, socioeconomic features (i.e., poverty, unemployment, educational attainment, income), density of primary care physicians, drug supply, and access to resources, such as drug treatment, health care, and prevention services (Altekruse, Cosgrove, Altekruse, Jenkins, & Blanco, 2020; Haffajee, Lin, Bohnert, Goldstick, & E., 2019; Hollingsworth, Ruhm, & Simon, 2017; Monnat, 2019; Rigg, Monnat, & Chavez, 2018). Further, there are distinctive patterns related to prescription opioid use and heroin use (Pear & Monnat, 2019).

To identify states that experience a heavy burden of disease from opioid-related deaths—and presumably have the most incentive to mount a systemic response to address OUD within their correctional systems—the study used a stratified sampling plan that included states with a disproportionate share of opioid overdose fatalities, as well as states with a large number of overdose fatalities. From a practical perspective, opioid-related overdose death is a standardized metric available across all states and, in the absence of resources to support a comprehensive survey of all U.S. states and territories, allows for selection of states that account for the vast majority of opioid overdose fatalities. Additionally, following the recommendations of key stakeholders who were consulted on the study methods, inclusion of states with very low rates of opioid overdose would lead to zero inflation of many responses. Thus states that had little need or incentive to respond and hence no or limited information on the effects of the opioid crisis on their prison systems were not included in the sampling frame.

The first stratum included states based on their per capita rate of opioid overdose deaths, thereby including states with overdose rates significantly higher than the national average. Next, states were included based on the absolute number of opioid-overdose fatalities, regardless of the per capita rate. Combined, these two criteria served as indicators of the disease burden related to OUD (Degenhardt et al., 2014).

Using data from the Centers for Disease Control and Prevention Multiple Causes of Death (MCD), the lead investigators identified that of the 48,476 opioid overdose deaths reported to CDC in 2017, 50% came from just 9 states. Another 23% of deaths were in an additional 14 states with crude opioid overdose death rates significantly above the national average. (Supplement A contains maps showing the crude rates of opioid overdose death and number of opioid-related deaths in 2017.) These 23 states selected for the targeted study sample represented 75% of all the opioid overdose deaths nationally and included a range of statewide populations: 10+ million (7), 2 to 9.9 million (10), and under 2 million (6).

### Measurement

Study measures were developed through a review of previous studies of OUD-related services and treatment within both prisons and jails, as well as consultation with correctional representatives and stakeholders. Information on interview development, including the sources consulted to derive the interview content, and the interview measures are contained in Supplement B. **Procedures.**

The lead investigators identified and worked with key stakeholders in the 23 targeted states (including state correctional administrators and medical officers) to identify appropriate contacts within each state. Once the appropriate prison system contact was identified, an interview coach was assigned to facilitate the interview process. State representatives were assured of confidentiality regarding their state’s participation in the study.

Study investigators met with each state contact to provide an overview of the study and interview procedures; at this time, administrators often designated a different individual or a multidisciplinary team to complete the interview. (Additional detail on respondent characteristics is included in the Results section.) The structured interview process required four to twelve weeks to finalize and were completed by returning the instrument, via phone, or a combination of the two. Phone interviews were also audio-recorded to ensure both quality of data and consistent coding.

Respondents were first asked about availability and implementation of services along the OUD service cascade within their “state prison system” generally and then asked to specify the “number of prisons” within the system that provided each service. The final sections asked about the barriers to implementation and training and technical assistance needs for providing MOUD more broadly within their system.

To aid in evaluating the quality of the data, the structured interview allowed participants to indicate whether their response was estimated or whether the information was either not accessible to them or was not collected. Depending on the range of services provided, as indicated by the respondent, the planned duration of the interview was between 30 to 90 min, and it was expected that answers to some questions might require input from multiple people/sources.

All interviews were conducted, keyed, and checked between March and December of 2019. The overall interview response rate was 91% (21/23). Interviews were reviewed for completeness, inconsistent responses, and legibility and, if necessary, clarifications were sought from the recording or respondent. Interviews were keyed and rekeyed in Qualtrics™. A reliability check of the two found 99.9% accuracy of data entry with most errors occurring in entry of open-text responses. For “other” response fields (e.g., types of barriers to each type of MOUD), two staff independently re-coded responses into existing categories; where they disagreed, the lead investigator reviewed the data and made the final decision.

### Statistical analyses

Data were analyzed with IBM SPSS™ Version 26, using the frequencies and descriptive procedures. Questions were eliminated from the analyses when data for 15% of states (4 or more) was either unavailable or not systematically collected. Where only 1 state was missing, the mean of the valid responses is reported (the equivalent of mean replacement). For some questions (e.g., route of administration, barriers to implementing across all prisons), there were skip outs, and the questions were asked only when applicable. These cases are clearly noted in the text and tables.

## Results

First, frequency distributions are presented for characteristics of the interview respondents and state prison systems represented in the study. Second, the percentage of systems in which at least one prison provides the indicated services as well as the percentage of prisons within state systems that provides the indicated services, where appropriate, are presented following the OUD cascade model: 1) screening for OUD; 2) withdrawal management; 3) availability and service delivery of MOUD treatment across medications and for each of the three types of medications; and 4) overdose prevention at re-entry. The final two sections examine barriers to MOUD in state systems where MOUD provision is not universal across prisons and resources needed to expand MOUD treatment and facilitate community linkages for the total sample.

### Characteristics of interview respondents and state prison systems

The respondents for the 21 states in the study sample were individuals with the most expertise and knowledge of their prison systems and/or were able to obtain the information necessary to complete the interview. Respondents were medical/behavioral health directors (67%), state commissioners (14%), deputies (14%), and policy/audit managers (5%) who had an average of 4.5 years (s.d. = 4.3) in their current position and 14.2 years (s.d. = 7.9) in the corrections field. In several cases, respondents identified one (*n* = 5) or two (*n* = 5) other people who assisted in gathering needed information. These additional respondents were program/service directors (73%), analysts (13%), commissioners (7%), and staff assistants (7%) who had an average of 5.2 years (s.d. = 4.0) in their current position and 14.3 years (s.d. = 10.6) in the corrections field.

Characteristics of the state prison systems for the total sample are shown in Table 1. Across the 21 state prison systems, there was a total of 583 prisons (ranging from 3 to 145 by state system) with 773,868 incarcerated individuals (ranging from 1133 to 162,729 by state system). Of the 583 prisons, 96% were state-run and 4% privately-run; 89% were male only, 9% female only, and the remaining housed both males and females.

**Table 1:** Characteristics of U.S. state prison systems

Characteristics of state systems (1+ prison) and state prisons that are:	% of state Systems with 1+ prisons (*n* = 21)	% of Prisons Across Systems (*n* = 583)
Managed		
State-run prisons	100%	96%
Privately-run prisons that report to the State Department of Corrections	29%	4%
Facility		
Male only	100%	89%
Female only	100%	9%
Male and Female	43%	3%

### Service provision across the OUD service Cascade

#### Screening for OUD

Of the 21 prison systems, 100% reported individuals are screened for OUD in one or more of their prisons (see Table 2) with screening most often conducted during the admission process (81%), after admission (33%), or on an as-needed basis through referral or upon request (38%). Among the 583 prisons, screening for OUD was conducted in 47% (*n* = 273) and most commonly using self-reported days of opioid use (99%), clinical assessments (96%), urine analysis (67%), and/or a standardized instrument (66%). The most common standardized instruments were the Texas Christian University Drug Screen (TCU DS; 39%), Addiction Severity Index (ASI; 21%), and Global Appraisal of Individual Needs (GAIN; 14%).

**Table 2:** OUD service cascade within state systems and state prisons in U.S

	% of state Systems with 1+ prison^\1^ (*n* = 21)	% of Prisons across systems (*n* = 583)
**Screening**		
Have protocol to identify people who likely have opioid use disorders (OUD)	100%	47%
Timing of screening as % of individuals (can be more than once)		
During Admission	81%	–
After Admission	33%	–
On an as-needed basis through referral or upon request	38%	–
Other	5%	–
Screening methods (of *n* = 273 prisons screening)		
Self-reported days of opioid use	–	99%
Clinical assessments	–	96%
Urine analysis	–	67%
Standardized instruments	–	66%
**Withdrawal Management**		
Standardized protocol to identify people who are or at risk of withdrawing from opioids	81%	43%
**MOUD Treatment**		
Provide at least one type of MOUD	100%	39%
Provide all 3 types	62%	7%
Provide no type of MOUD	0%	61%
Population that the state system provides MOUD to:		
People close to release or with a release date	81%	–
Pregnant women	81%	–
Those admitted on any type of MOUD	38%	–
Court-ordered MOUD	29%	–
Anyone with an OUD	29%	–
Other	24%	–
Only pregnant women	14%	–
Only those admitted on any type of MOUD	0%	–
No one	0%	–
**Re-Entry**		
Provide incarcerated individuals training how to use naloxone to reverse an overdose	60%	27%
Provide naloxone kits to individuals at release	48%	23%

^\1^Based on percent of states with 1 or more prisons

### Withdrawal management

In 81% of state prison systems, a protocol for identifying current and potential withdrawal from opioids is utilized in one or more prisons and 43% have a protocol for withdrawal management (see Table 2). Medication for withdrawal management is provided in 41% of the 538 prisons (*n* = 238) with the most common being benzodiazepines (61%), buprenorphine (53%), methadone (28%), Lofexidine (5%), and other medications like clonidine or gabapentin (51%).

### Availability of MOUD treatment by type of medication

While 100% of the state prison systems reported 1 or more of their prisons provide at least one type of MOUD, 62% of the systems reported 1 or more of their prisons provide all three types of MOUD. Despite this, only 7% of the 538 prisons across these 21 systems provide all three types; 39% provide at least one type, and 61% do not provide any type of MOUD.

There was also considerable variation regarding the criteria used by state systems to determine an individual’s eligibility to receive MOUD treatment (see Table 2). Even though a system may indicate the provision of a particular medication, availability or accessibility is often limited. To better understand how access may be limited, respondents were asked to indicate the necessary conditions and subpopulations for which MOUD treatment is available in one or more of their prisons. Responses from systems indicated that MOUD treatment is available to: individuals close to release or with a release date (81%); pregnant women (81%); individuals admitted on MOUD (38%); individuals court ordered to be on MOUD (29%); any individual with an OUD (29%); and only pregnant women (14%). The next three sections report data on the availability and accessibility of each of the three types of MOUD.

### Buprenorphine availability and service delivery models

In 91% (19/21) of state prison systems, treatment with buprenorphine is available to individuals with OUD in one or more of their prisons and is accessible to a subset of individuals in 15% of prisons across these systems (see Table 3). Specifically, state systems provided buprenorphine treatment to pregnant women (81%, 38% provided only to pregnant women), people who were receiving buprenorphine when admitted to prison (52%), individuals close to release or with a release date (38%), and others, excluding pregnant women and those already receiving it (38%).

**Table 3:** To whom and at what point MOUD is available by type of medication

MOUD availability within state prison systems	Buprenorphine	Methadone	Naltrexone
**Available in the state system (*n*** **= 21 states)**	**91%**	**91%**	**86%**
Availability in prisons (*n* = 583 prisons)	15%	9%	36%
**Medication provided to (*n*** **= 21):**			
Pregnant women	81%	86%	–
*Pregnant women only*	*38%*	*57%*	*–*
People who were already receiving the medication when admitted to prison	52%	38%	38%
Individuals being released	38%	24%	86%
Others (pregnant women those entering prison on it)	38%	29%	43%
**Medication used for (*n*** **= 21):**			
Continuity of care	91%	86%	38%
Induction and treatment during confinement	29%	33%	48%
Managing acute or chronic pain	24%	20%	–
Pre-release induction	38%	24%	86%
**Medication provided by (*n*** **= 21):**			
External contracted vendors on-site	52%	48%	57%
External contracted vendors via transportation off site	14%	52%	0%
Prison health care teams	14%	10%	19%
Combination/Other	14%	5%	10%

Access across systems also varied in regard to where along the opioid cascade the state prison system provided buprenorphine. It was provided for continuity of care in one or more of the prisons in 91% of the 21 systems; for pre-release induction (38%); to induct and maintain during confinement (29%); and to manage acute or chronic pain (24%).

In terms of service delivery, state systems provided buprenorphine treatment onsite through external contracted vendors or waivered physicians (52%), off-site by medical transport (14%), onsite by prison healthcare teams or waivered physicians (14%), or a combination of the above (14%).

### Methadone availability and service delivery models

In 91% of state prison systems, methadone is available to individuals with OUD in one or more of their prisons (see Table 3). Methadone treatment is accessible to a subset of individuals in 9% of the 583 prisons across these systems. Specifically, state systems provide methadone treatment to pregnant women (86%; 57% provide only to pregnant women), people already being treated with methadone at admission (38%), people close to release or with a release date (24%), and others (29%).

Like buprenorphine, methadone is provided at various points along the OUD service cascade. It was provided for continuity of care in one or more prisons in 86% of the 21 state systems; to induct and maintain during confinement (33%); for pre-release induction (24%); and to manage acute or chronic pain (20%). State systems most often provide methadone treatment off-site through medical transport (52%). Close to half provide it onsite by an external agency (48%) or onsite by a prison healthcare team under a certified Opioid Treatment Program (10%).

### Naltrexone availability and service delivery models

In 86% (18/21) of state prison systems, injectable or oral naltrexone is available to individuals with OUD in one or more of their prisons, and is provided in 36% of the 538 prisons across these systems (see Table 3). As with the other types of MOUD treatment, naltrexone is available to a subset including: individuals close to release or with a release date (86%); others, excluding pregnant women and those already being treated with it (43%); and people already being treated with it at admission (38%).

Naltrexone is most frequently administered at pre-release induction (86%), but is also used to induct and treat during confinement (48%), and to provide continuity of care (38%). State systems provide naltrexone treatment onsite by external/contracted vendor (57%), onsite by prison healthcare team or waivered physician (19%), or a combination of both (10%).

### Overdose prevention at re-entry

In 60% of the 21 state prison systems, naloxone training is provided to individuals in one or more of their prisons and close to half (48%) of these systems provide naloxone kits to individuals upon release in at least one of their prisons. Yet naloxone distribution at re-entry is not comprehensive across prisosns; across the 583 prisons, 27% provide naloxone training to individuals and 25% provide naloxone to individuals upon release.

### Barriers to MOUD delivery

Respondents were asked about the barriers to providing each form of MOUD within their state prison system when it was not available to everyone with an OUD in all prisons in their system, from a list of 10 barriers that limit the availability of treatment (see Table 4).

**Table 4:** Barriers to MOUD access by type of medication

Barriers to providing MOUD to everyone with an OUD in every prison	Type of Medication for OUD (MOUD)^**\1**^		
	Buprenorphine (***n*** = 18)	Methadone (***n*** = 18)	Naltrexone (***n*** = 14)
The state lacks funds needed for the medical and clinical staff to support the provision of the medication	67%	67%	43%
The state lacks funds needed for resources to prevent diversion	61%	44%	14%
The state lacks funds to pay for the medication	50%	50%	71%
The state prefers more of an abstinence-based approach to treatment	39%	39%	14%
Regulations make it difficult to provide the medication	33%	61%	14%
Lack of access to training or technical assistance on its use	22%	11%	21%
Inadequate reimbursement rate	17%	6%	0%
There are not enough community providers of the medication to work with individuals following release	11%	33%	29%
The facilities lack necessary relationships with community MOUD providers	11%	28%	29%
People confined to state prisons do not want this type of medication	6%	6%	36%
Other	11%	28%	21%

^\1^Column samples sizes excluded states in which each medication is already available to everyone with an OUD in all of the prisons in that state system

With regard to buprenorphine, the top three barriers included: 1) the state lacks funds for the necessary medical/clinical staff (67%); 2) the state lacks funds needed to prevent diversion (61%); and 3) the state lacks funds to pay for the buprenorphine (50%). Approximately one-third of prison systems also indicated the state prefers a more abstinence-based approach to treatment (39%) and regulations make it difficult to provide this medication (33%).

The top three barriers that prevent systems from providing methadone in all prisons include: 1) the state lacks funds for medical/clinical staff (67%); 2) regulations make it difficult to provide methadone (61%); and 3) the state lacks funds to pay for the methadone (50%). Over one third also cited the lack of funds needed to prevent diversion (44%); the state prefers a more abstinence-based approach to treatment (39%); and there is an inadequate number of community providers to work with individuals following release (33%).

Two of the top three barriers to state systems providing naltrexone were the same as the other treatments: lack of funds to pay for naltrexone (71%) and lack of funds to pay for medical/clinical staff (43%). The third, however, was unique: people confined to state prisons do not want naltrexone (36%). No other barrier to provision of naltrexone was endorsed by 30% or more of the prison systems.

### Resources needed to expand MOUD treatment and facilitate community linkages

Both internal and external factors have been demonstrated to be important to the implementation and scaling of evidence-based practices (Watson et al., 2018). Consistent with this, the study identified a number of MOUD implementation needs existing both within and outside of prison walls. Many of these needs overlap with those identified as MOUD implementation determinants in prior research (Knudsen, Abraham, & Oser, 2011; Scorsone, Haozous, Hayes, & Cox, 2020), while others were identified by respondents during the pilot phase.

At the time of these interviews, only 7% of prisons in these state systems provided all three types of MOUD treatment to individuals with OUD. To help advance the science of implementing MOUD in prisons, respondents were asked about their needs related to expanding MOUD and to provide successful linkages to MOUD in the community following release. Table 5 includes 40 items organized around 5 themes in which respondents indicated the need for help: a) addressing stigma, b) funding, c) education regarding OUD, addiction, interface of OUD and justice, and benefits of MOUD, d) logistical and programmatic needs within the walls, and e) re-entry support. Respondents endorsed the needs currently encountered by their systems.

**Table 5:** Resources needed to expand MOUD and facilitate community linkages

	Percent
**Help addressing stigma and negative attitudes towards MOUD** **Additional funding for**	91%
Medication	100%
Resources needed to prevent diversion	100%
Clinical staff to administer and monitor MOUD	95%
Transportation to MOUD	81%
MOUD in the community	81%
**Education**	
Probation/Parole staff	86%
State/local politicians and other key stakeholders	81%
Inmates	76%
Correctional staff	76%
Clinical staff/physicians	76%
General community	71%
Pregnant w omen	71%
Judges	67%
DOC administrators	62%
Churches	62%
District attorneys	52%
Other	33%
**Inside the walls**	
Logistics	
Minimize diversion	91%
Establish systems to screen people	86%
Implement ECHO/Telemedicine	67%
Become licensed OTP	62%
Test for illicit drug use	57%
Obtain waivers	52%
Medication related	
Add medical staff	91%
Match needs with type of MOUD	81%
Arrange dosing of methadone and/or bup by community program	76%
Switch between types of MOUD	71%
Supervise oral administration of MOUD	62%
Administer, monitor, store medication	62%
Establish MOUD in pregnancy program	57%
MOUD administration	52%
**Re-entry support**	
Funding for MOUD post-release	95%
Same-day access to MOUD	86%
Provision of MOUD continuity of care upon re-entry into communities without MOUD	86%
Access to employment	86%
Solutions to regulatory, insurance, or managed care limits for post-release continuation of MOUD	81%
Access to sober-housing	81%
Reactivation and/or application for Medicaid to help with re-entry	81%
Access to ID	57%
MOUs for re-entry services	48%
Strategies for building community partnerships and establishing agreements for MOUD post-release	48%

To expand MOUD treatment across these systems, there is a clear need for help addressing stigma associated with OUD and its treatment. Specifically, 91% of respondents indicated they need help addressing stigma and negative attitudes towards MOUD treatment. Secondarily, given the costs associated with delivering MOUD in prisons, it was not surprising that 81% to 100% endorsed needs for additional funds to support: a) cost of medication (100%), b) prevent diversion (100%), c) clinical staff to administer and monitor MOUD (95%), d) transportation to MOUD (81%), and e) MOUD in the community (81%).

In addition, over two-thirds of respondents indicated that general education regarding OUD, addiction, the interface of OUD and the justice system, and the benefits of MOUD must be provided to: a) probation and parole staff (86%), b) state and local politicians, and other key stakeholders (81%), c) incarcerated individuals, correctional, clinical and medical staff (76% each), d) general community and pregnant women (71% each), and e) judges (67%).

With regard to logistical barriers inside the walls, over two-thirds of state systems indicated the need for resources to minimize diversion (e.g., scanners, staff) (91%), help establishing systems for OUD screening (86%), and training on Extension for Community Healthcare Outcomes (ECHO) and telemedicine (67%). Respondents also need resources to add medical staff (91%), information on how best to match the needs of individuals with the appropriate type of MOUD (81%), strategies for arranging MOUD dosing by community providers (76%), and switching between different types of MOUD (71%).

In terms of facilitating successful community linkages, there was considerable discussion during the interview process about the ethical and logistical challenges of linking individuals to MOUD in the community following release. Given that individuals are often incarcerated in geographic areas far from their communities, the challenges to provide successful linkages were numerous and varied. Funding for MOUD post-release was nearly universally endorsed (95%). Moreover, ensuring continuity-of-care at release was a critical concern, with 86% of respondents endorsing both the need for same-day access to MOUD at re-entry and the need to provide MOUD when individuals are released to communities that lack a MOUD provider. Relatedly, 81% endorsed needing help with regulatory, insurance, or managed care limits for post-release continuation of MOUD, including obtaining Medicaid for those being released. The majority also felt that access to employment and sober housing (86% and 81%, respectively), were critical to the success of those returning to the community.

## Discussion

This study was conducted to obtain detailed information regarding the ways in which prisons systems are currently addressing the impact of the opioid epidemic. Unlike prior studies that surveyed medical directors of all state prison systems, this study targeted prison systems in states that are most severely impacted by the opioid epidemic. Interviews focused on delivery of services across the criminal justice OUD service cascade, barriers to providing MOUD, and the resources, financial support, education, and training necessary to expand the availability and accessibility of MOUD. The interview also included a wider range of questions regarding the expanded list of FDA-approved medications for OUD (i.e., methadone, buprenorphine, and naltrexone), as well as the use of medications for withdrawal management and overdose prevention.

### Provision of MOUD within state prison systems

Overall, the study found that nearly two-thirds (61%) of all prisons in the state systems included in this study do not provide any form of MOUD. Moreover, although a majority provide it to pregnant women, less than one third provide it unconditionally to anyone with an OUD. When examined within the framework of the OUD services cascade within prisons, the study findings yielded a complex picture of MOUD-related services across the state prison systems. The majority of systems need help establishing systems for OUD screening and medication management, such as matching an individual’s needs with different types of MOUD or changing from one type to another. Within these state systems, less than half of the prisons utilize a protocol for withdrawal management and only 41% provide medication for withdrawal management.

Although the current study demonstrates more forms of MOUD are available across state prison systems than in prior studies, many of the previously identified barriers continue to exist (Belenko, Hiller, & Hamilton, 2013; McKenzie, Nunn, Zaller, Bazazi, & Rich, 2009). Moreover, there are differences in how the types of MOUD are implemented and for whom, and the challenges and barriers encountered regarding each. For example, the majority of systems need guidance when arranging for methadone and/or buprenorphine by community programs during confinement.

Treatment with buprenorphine is widely available across state prison systems. Over half (52%) of state systems dispense buprenorphine to individuals who are already being treated with this medication at the time of their admission (i.e., continuing care). This may reflect greater access to treatment with buprenorphine in community opioid treatment programs—as well as from private physicians—that has occurred in the past decade (Alderks, 2017), and is a significant shift from prior studies. Yet its use is restricted to a relatively small proportion of the prisons within these state systems (15%), suggesting that most states have opted to specialize treatment with buprenorphine in a few facilities within their systems. Such an approach may have organizational and economic efficiencies, relative to a system-wide approach to developing MOUD capacity across state prisons, and no conclusions can be drawn from the current study about the adequacy of MOUD provision in these different approaches. Additional research is needed to assess the outcomes and cost effectiveness of limiting the provision of buprenorphine to a sub-set of facilities versus implementing it system-wide, as well as the barriers to expanding buprenorphine beyond a few select facilities.

Treatment with methadone is also widely used across state prison systems although its use remains largely restricted to specific subgroups of individuals, particularly pregnant women and those who were receiving it at the time of admission (continuing care). Moreover, few prisons within these systems are equipped to provide methadone (9%). Regulatory barriers continue to impede the use of methadone within state prisons, resulting in prisons contracting with outside providers to dispense it on-site, or prison staff transporting individuals to off-site facilities.

As with the other forms of MOUD, naltrexone is broadly available across most state prison systems; unlike the other forms, however, it is also more widely available within the prisons in these systems (36%). In addition to providing naltrexone to individuals who were receiving it at the time of admission, it is typically dispensed to individuals prior to their discharge. Unlike the other forms of MOUD, however, use of naltrexone is impeded by an aversion among individuals with OUD. Other studies have found this aversion to naltrexone stemmed from a lack of information about the medication, belief that it is a “crutch,” or a fear that it would precipitate withdrawal (Marcus et al., 2018). However, a study conducted with individuals incarcerated in Norway found that many rejected naltrexone as a treatment option because they preferred abstinence, since they had already gone through withdrawal (Puglisi, Bedell, Steiner, & Wang, 2019). These findings suggest that greater access to naltrexone as a treatment option in prison needs to be coupled with education about its use and benefits in averting relapse. Indeed, approximately three quarters of state representatives identified the need to educate incarcerated individuals about OUD and use of MOUD as necessary for expanding its use within their systems.

Continuity of care for individuals already being treated with MOUD at entry was most often cited as the reason for providing buprenorphine and methadone, and was the second most-cited reason for providing naltrexone (following its use prior to release). However, since only a subset of prisons provided these medications, most individuals being treated with MOUD when entering prison do not receive continuing care while incarcerated. Forced detoxification can have deleterious effects, create an aversion to these medications, and heighten the risk of relapse (Aronowitz & Laurent, 2016; Fu, Zaller, Yokell, Bazazi, & Rich, 2013; Mitchell et al., 2009; Rich & McKenzie, 2015). Policies aimed at improving provision of MOUD to justice-involved individuals can prioritize continuing care at prison entry so individuals are not suddenly unable to continue their treatment.

### Re-entry services

The last step in the criminal justice OUD service cascade, re-entry, was also of concern to respondents. They highlighted the need for increased availability of MOUD treatment in the community, transportation to treatment following release, and access to same-day treatment and sober housing. Additionally, a large majority of respondents endorsed helping individuals to apply for Medicaid following their release as a priority. States that have implemented the Medicaid expansion have higher rates of criminal justice referrals to treatment with MOUD, compared to non-expansion states; however, individuals with OUD who are referred from the criminal justice system have lower rates of treatment with MOUD compared to individuals referred from other sources, showing continuing disparity in access to MOUD even in states that have enacted the Medicaid expansion (Khatri, Howell, & Winkelman, 2021).

Given these challenges to accessing treatment with MOUD following release, many respondents discussed the ethical issues of providing MOUD treatment to individuals while incarcerated knowing it would be difficult for them to access treatment upon release due to the lack of or linkages to community treatment providers. Building relationships with community providers would improve reentry success. Establishing linkages between these community-based treatment providers and correctional systems staff (parole officers in particular) could provide opportunities for both to learn more about the needs of re-entering populations, and improve the coordination of community re-entry procedures across systems (Monico & Mitchell, 2016).

Lastly, overdose prevention is an essential component to re-entry, given the high risk of overdose following release, as noted previously. However, only half of the systems dispensed naloxone to individuals upon release from prison. Although take-home naloxone programs are in the early stages of implementation, one rigorous study of the national naloxone take-home program in Scotland provided evidence of reduced post-release mortality from opioid overdose (Bird, Fischbacher, Graham, & Fraser, 2015; Bird, McAuley, Perry, & Hunter, 2016; Horsburgh & McAuley, 2018). A critical component for the reduction of post-release mortality from opioid overdose is linkage of individuals to community-based programs they are likely to use, such as drug treatment, harm reduction, or other health and social services (Zucker, Annucci, Stancliff, & Catania, 2015). Such programs can provide access points for naloxone distribution and ongoing prevention interventions (Grella et al., 2021).

### Barriers to provision of MOUD and needed education and resources

To enable expansion of MOUD within their systems, large majorities of the respondents identified needs for training to prevent MOUD diversion, screening for OUD, appropriate selection of medication, and coordination with community providers. Professional prison staff have not previously been required to have the knowledge necessary to support an OUD-certified treatment program. Hence, training and technical assistance are essential to expand knowledge of best practices and progress with regard to MOUD treatment. There is a need—perhaps national in scope—to provide training material targeting prison professionals, along with technical assistance for implementation.

With over one third of systems identifying a state preference for abstinence-based treatment as a barrier to adoption of MOUD, greater education for stakeholders of the benefits of MOUD as an evidence-based treatment may help expand adoption. This could be accomplished at least two ways. The first is to incentivize prison systems to adopt MOUD treatment using federal funds in the form of new targeted grants for corrections or through modifying existing federal grant programs to place more emphasis on providing MOUD in correctional settings. The second is to provide ongoing national programs to educate leadership and correctional line staff about the effectiveness of MOUD treatment. This could be achieved by enlisting the support of professional organizations (e.g., American Society of Addiction Medicine, NCCHC) to apprise correctional leadership of how MOUD treatment can improve the health and safety of incarcerated individuals, enable their smooth transition back into the community, and reduce their risks of recidivism, relapse, and death.

Overall, lack of funds was the most frequently cited barrier to implementing all types of MOUD, either with regard to funding for medical staff or to cover cost of the medication, consistent with other studies of implementation of MOUD within correctional settings (Ferguson et al., 2019). However, unique issues were cited as barriers for each of the three types of medication. Funding to address diversion was a top concern regarding buprenorphine, regulatory prohibitions were more often cited as a barrier to providing methadone, and a lack of preference for its use among incarcerated individuals was prominent for naltrexone. To a lesser extent, respondents identified the lack of community linkages necessary to support a comprehensive MOUD delivery system, as well as the continuing abstinence-based treatment approaches that prevail in some areas. Awareness of these commonly-cited potential barriers is key to guiding policies and initiatives designed to expand MOUD use in state prisons.

In 2017, the U.S. government declared the opioid epidemic is a nationwide public health emergency (Assistant Secretary for Preparedness and Response, 2017), and funded efforts to address it (Galvin, 2017; National Conference of State Legislatures, 2020;). The twenty-first Century Cures Act and the Comprehensive Addiction and Recovery Act—both signed into law in 2016—along with subsequent legislation, have allocated billions of dollars to help states with the opioid crisis. While this funding has been used to expand access to treatment, recovery, and prevention services in communities across the U.S., there is little evidence that substantial additional funding has reached state correctional systems. Finally, federal funding, specifically tailored toward correctional systems, and in coordination with the already-allocated community-level funding, would significantly help close the resource gap they face. Such appropriations, alongside additional federal fiscal relief to states, could dramatically help address the ongoing opioid epidemic and prevent cuts to resources that support the OUD services cascade within state prison systems.

In addition to expanded funding, the majority of respondents indicated the need for education on OUD, addiction, MOUD, and stigma for a wide range of audiences “outside the walls,” including judges, clinical and medical staff, probation, parole, churches, and the general community. A recent national population survey of beliefs about opioid addiction found that stigma associated with OUD was positively associated with support for discriminatory actions against people with OUD in areas such as education, health care, employment, and housing; it was negatively associated with support for policies favorable to people with OUD, such as expanding insurance coverage for treatment of OUD, access to Naloxone, and government funding for OUD treatment (Adams et al., 2021; Taylor et al., 2021). Moreover, this pervasive stigma regarding OUD is embedded in social policies that favor criminalization of individuals with OUD, leading to their high rates of incarceration (Tsai et al., 2019). Hence, social policies that shift the focus from criminalization to public health inteventions for OUD (Wakeman & Rich, 2015), along with training, education and community-wide efforts at stigma reduction, must be part of a concerted strategy to expand MOUD provision to individuals in the community, including those who are involved in the criminal justice system.

### Study strengths and limitations

This study was an in-depth examination of specific issues regarding the implementation of MOUD in state prisons by concentrating on states that have been highly impacted by the current opioid epidemic, either by virtue of a large number of deaths or by a high rate of deaths per capita. This approach identified states most likely to be providing MOUD within their prison systems and hence able to respond to detailed questions on its use. However, the sampling strategy did not allow for simple comparisons with findings from prior studies in which all states were surveyed or studies that targeted specific states. A comprehensive survey of all U.S. states and territories, while requiring more resources, may have identified emergent needs in states where the effects of opioid overdose were increasing. Nevertheless, the findings from this study, which included states representing 75% of opioid deaths nationally, demonstrate the need for a national effort to support the OUD services cascade to target hardest hit areas first and to monitor for escalation of opioid overdose rates and their effects on state prison systems in areas that have not yet mounted a response.

In addition, although the study employed a set of quality assurance checks, the responses are self-reported by state correctional system representatives. An attempt was made to monitor the quality of data by asking respondents to indicate if they were estimating the data, did not have access to it, or did not collect it; variables that were deemed to be most problematic in terms of data quality were not included in this analysis. Similarly, questions missing a large amount of data were not included in the analyses. This occurred most often in questions regarding the characteristics of the prison population and receipt of services, which were missing data from one half to two thirds of respondents. The 21 respondents were asked to describe the number and characteristics of their prisons in aggregate on multiple questions; however, data by prison that would allow for more detailed analyses using prison as the unit of analysis is not available.

Lastly, it was evident that the state prison systems are currently in flux. Some respondents noted they were planning to broaden their capacity to provide MOUD more uniformly across their system or, alternatively, to concentrate limited resources in fewer prisons that are specialized for providing treatment for OUD. Many were in various stages of developing their OUD treatment capacity, either building on existing SUD treatment programs or establishing new prison-based treatment programs. Thus, the findings reported in this paper shed light on the status of MOUD provision at a specific point in time. Evolving strategies are needed within a “dynamic” approach to drug policy, in which responses to population indicators of need are fluid over the course of a drug epidemic (Caulkins, 2006). Moreover, the study addresses state systems’ capacity to provide MOUD and other services for OUD along a cascade of care, but not the quality, effectiveness, or adequacy of these services or organizational strategies for their delivery. Future research is needed to assess the effectiveness of different strategies for implementing MOUD within state systems, such as comparisons of broadly distributed capacity versus specialization of resources within a sub-set of prisons. Lastly, the interviews were conducted prior to the onset of the COVID-19 pandemic, which had a dramatic effect on correctional facilities in the U.S. (Mukherjee & El-Bassel, 2020), and may have altered the implementation of MOUD treatment within these systems.

## Conclusion

Study findings yield a complex picture of how, when, and to whom MOUD is provided to individuals with OUD across prison systems in states most heavily impacted by opioid overdose in the United States. Moreover, the findings suggest strategies and policies to promote the provision of MOUD to better address the needs of incarcerated individuals with OUD through expansion of education and training, screening and assessment, resources, and clinical capacity. Although this study identified the lack of funding as a significant barrier to the successful implementation of MOUD within state prison systems, recent federal funding initiatives provide potential resources. Yet, funding for additional resources, training, and education is not sufficient; critical to implementing MOUD within state prisons is a commitment to addressing the effects of the opioid crisis across leaders in criminal justice systems, health care, government, and community partners (Ferguson et al., 2019). Leaders across these systems can utilize the findings from the current study to advance policies to support the expansion of MOUD capacity to address the needs of individuals with OUD in state prisons.

## Supplementary Information

**Additional file 1. **Number and Rate of Opioid-Related Deaths by State: 2017.

**Additional file 2. **Instrument Development and Variable Domains for JCOIN Prison Interview.

## Data Availability

The datasets used and/or analyzed during the current study are available from the corresponding author on reasonable request.

## Abbreviations

ACA: American Correctional Association
ASI: Addiction Severity Index
CDC: Centers for Disease Control
CJ-DATS: Criminal Justice Drug Abuse Treatment Studies
DOC: Department of Corrections
ECHO: Extension for Community Healthcare Outcomes
FDA: Food and Drug Administration
GAIN: Global Appraisal of Individual Needs
HIV: Human immunodeficiency virus
MCD: Multiple causes of death
MOUD: Medication for opioid use disorder
NCCHC: National Commission on Correctional Health Care
NSDUH: National Survey on Drug Use and Health
OR: Odds ratio
OTP: opioid treatment program
OUD: Opioid use disorder
RCT: Randomized controlled trial
SAMHSA: Substance Abuse and Mental Health Services Administration
TCU DS: Texas Christian University Drug Screen
