# Supplementary material for: The impact of the opioid crisis on U.S. state prison systems
Source: Health Justice. 2021 Jul 24;9:17. doi: 10.1186/s40352-021-00143-9 (PMC8310396; doi:10.1186/s40352-021-00143-9)
Supplement: Supplementary file 1 — Additional file 1. Number and Rate of Opioid-Related Deaths by State: 2017. [file 40352_2021_143_MOESM1_ESM.pptx]

## Slide 1
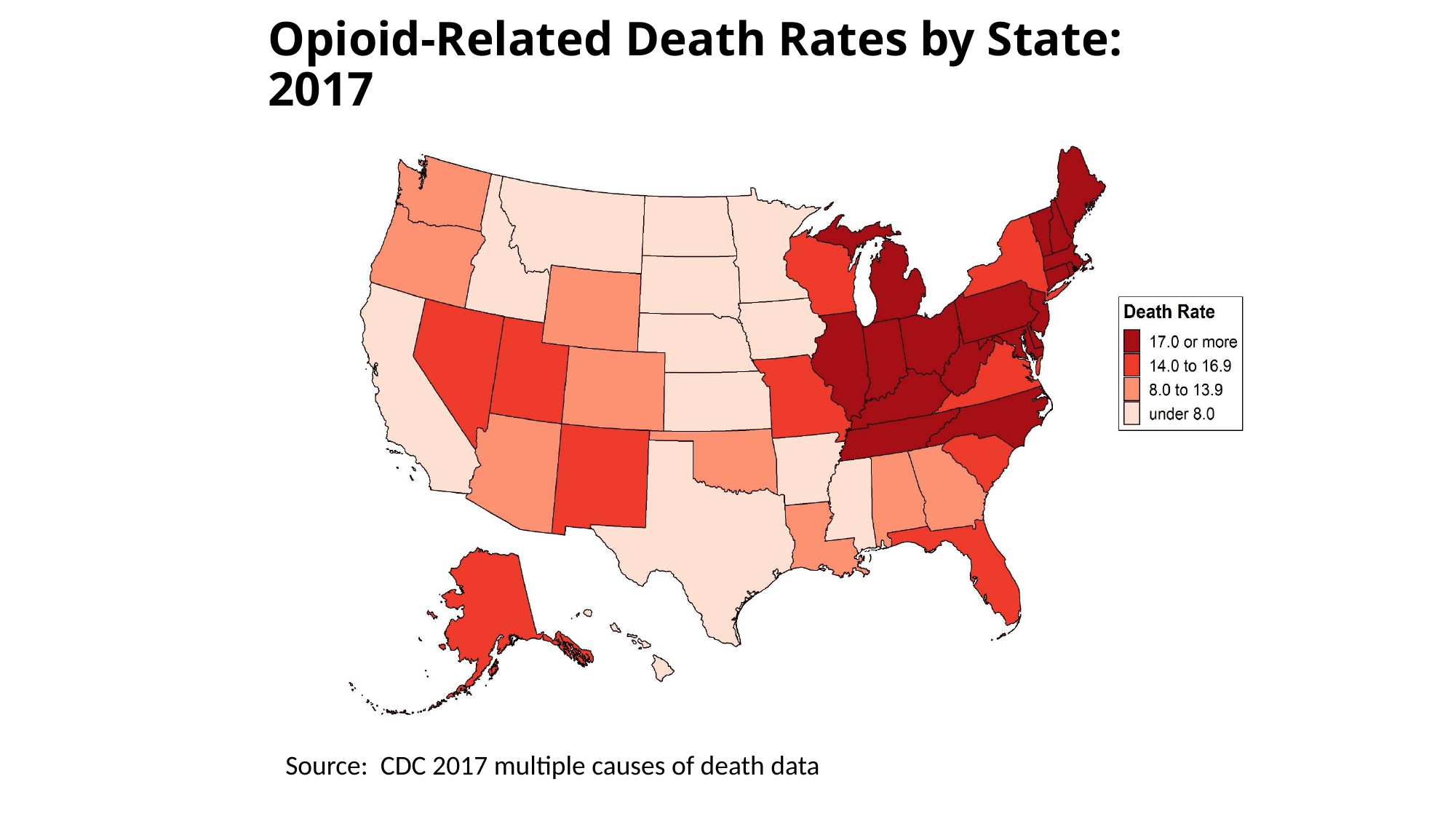

# Opioid-Related Death Rates by State: 2017
Source: CDC 2017 multiple causes of death data

## Slide 2
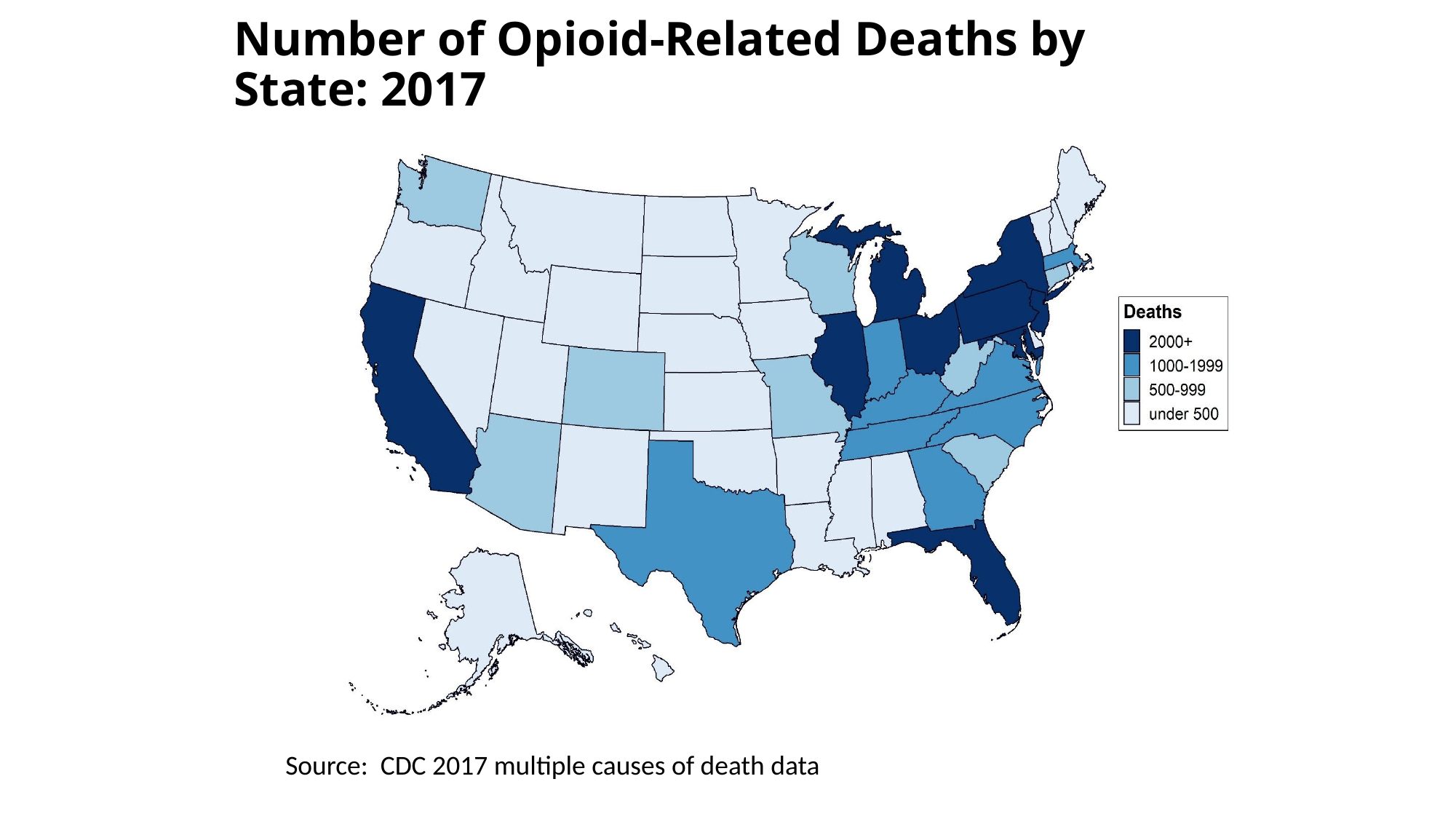

# Number of Opioid-Related Deaths by State: 2017
Source: CDC 2017 multiple causes of death data
